# Foliar biostimulant boost physiological resilience and reproductive growth, increasing cotton yield

**DOI:** 10.3389/fpls.2026.1852624

**Published:** 2026-07-03

**Authors:** Luiz Gustavo Moretti, João William Bossolani, José Roberto Portugal, Francesco Magro, Eleonora Perucco, Giacomo Masetti, Carlos Alexandre Costa Crusciol

**Affiliations:** 1 Embrapa Cerrados, Planaltina, Brazil; 2 Department of Crop Science, College of Agricultural Sciences, São Paulo State University (UNESP), Botucatu, Brazil; 3 Sofbey SA by Sipcam Oxon, Mendrisio, Switzerland

**Keywords:** abiotic stress, biostimulants, cotton, physiological stimulus, plant resilient

## Abstract

**Introduction:**

Cotton is a crop of major economic importance, often cultivated under environmental conditions that expose plants to abiotic stresses such as heat and water deficit, which negatively affect physiological performance and yield. Biostimulants have emerged as promising tools to enhance plant resilience and productivity under such conditions.

**Methods:**

This study evaluated the effects of foliar application of a novel biostimulant “SB”, based on a Glicoligno Lipidic Complex, at different dose rates on antioxidant metabolism, photosynthetic performance, agronomic traits, and boll yield of cotton grown under central Brazil conditions. The treatments promoted significant improvements in physiological efficiency, particularly at higher application rates, with the 2.0 L ha^-^¹ dose showing the most pronounced responses.

**Results and discussion:**

Foliar application of the novel biostimulant SB (Glicoligno Lipidic complex) significantly improved physiological and agronomic performance of cotton grown under central Brazil conditions. SB enhanced net photosynthesis (A) by up to 15.3%, and water use efficiency (WUE) by 12-20.5% (*p* > 0.1) across the evaluated cultivars and application times. Also, it increased RuBisCO activity by 9–22.4%, and improved chlorophyll and carotenoid contents (*p* > 0.05) by up to 25.2% and 16.3%, respectively. Antioxidant responses were modulated, with hydrogen peroxide (H_2_O_2_) and malondialdehyde (MDA) levels reduced (*p* > 0.1) by up to 12.4% and 14.3%, respectively, indicating reduced oxidative stress. These physiological benefits translated into higher reproductive success, with number of bolls per plant increased by approximately 30% in cultivar C_1_ and boll weight increasing by 6–6.3% in C_2_. Overall fiber seed yield was enhanced (*p* > 0.05) by 6.9% in C_1_ (*p* > 0.1) (cultivar 1; FM 911GLTP) and 5.6% in C_2_ (cultivar 2; FM974 GLT). The results highlight SB as an effective foliar biostimulant that improves photosynthetic efficiency, stress tolerance, and yield formation, supporting cotton productivity under variable field conditions.

## Graphical Abstract

**Figure:**
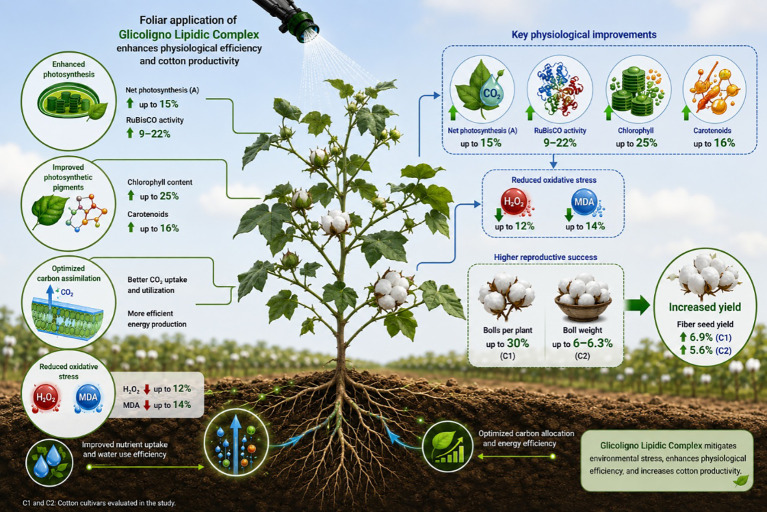

## 1 Introduction

Cotton (*Gossypium hirsutum* L.) is a globally important crop, providing natural fiber, oil and protein used in human and animal nutrition (Aparecido et al., 2020; Rani et al., 2025). Cotton is a perennial species that is cultivated as an annual crop; thus, depending on the cultivar, edaphoclimatic conditions, and irrigation management, its cycle can last from 140 to 180 days, divided into stages of emergence, vegetative growth, square formation, flowering, and maturation (Viveiros et al., 2024, 2025). Climatic conditions vary greatly during this long period in the field and can include extended periods of drought, particularly in Brazil, where cotton is typically grown as a second crop following soybean (Rani et al., 2025). Moreover, since the 1990s, cotton production in Brazil has shifted to the Cerrado region in response to severe yield declines due to pests and diseases in traditional producing regions. While plant breeding efforts have developed cultivars better adapted to the specific conditions of the Brazilian Cerrado´s low-fertility, acidic soils, extended dry periods, and high temperatures (Pavinato et al., 2009; Galindo et al., 2017), rapid climate change is subjecting cotton crops to increasingly severe environmental stresses, primarily abiotic stresses such as droughtand extreme temperatures (Momesso et al., 2022).

Abiotic stressors induce chemical and physical changes in plants that negatively affect plant physiology and metabolism, reducing growth, development, and yield (Bossolani et al., 2020; Fonseca et al., 2022). A central process of abiotic stress in plants is the induction of metabolic responses linked to oxidative stress. The excessive production and accumulation of reactive oxygen species (ROS) cause cellular damage, often irreversible, via lipid peroxidation in membranes (Rodrigues et al., 2021b). If not neutralized, ROS can inhibit development through mechanisms such as leaf abscission, altered root gravitropism, and, notably, damage to the photosynthetic system, all of which directly impair crop productivity (de Souza et al., 2025a; de Souza et al., 2025b).

New technologies focused on plant physiology and field management practices are being developed to alleviate the effects of abiotic stress and oxidative stress under adverse environmental conditions (Yakhin et al. 2017). One promising strategy is the application of biostimulants derived from natural raw materials such as seaweed extracts, humic and fulvic acids, amino acids, peptides, microorganisms, plant hormones, polysaccharides, phenolic compounds, and chitosan (Moretti et al., 2024; Ali et al., 2021). These resources, which are rich in macro- and micronutrients, vitamins, biopolymers, and bioactive molecules, have been shown to modulate plant physiological processes, promoting growth, stress tolerance, and productivity (Moretti et al., 2026c). Biostimulants can enhance nutrient uptake and utilization, increase antioxidant activity, and protect plants from photooxidative stress, resulting in improved root and shoot development, photosynthetic stability, and yield even under unfavorable climatic conditions (Moretti et al., 2026c, b, a).

However, there is still lack of information regarding the mode of action on plant´s physiology mechanisms due to the use of novel biostimulant formulations in field conditions and commercial crops management, especially for those related to complex molecular structures products (Kutasy et al. 2025). Additionally, it is important to build a solid and deep comprehensive understanding on how antioxidant metabolism and photosynthetic parameters respond as an integrated system according to this biostimulant application (Oliveira et al., 2022).

Before biostimulants can be widely applied in commercial cotton production, a comprehensive understanding of cotton cultivation under inherently stressful growing conditions is needed, particularly its adaptive responses to climate change, the underlying mechanisms, and the impact of specific types of biostimulants on responses and productivity gains and losses (Wozniak et al., 2020; Silva et al., 2020). Thus, this study represents a pre-market validation aimed at investigating the effects of different dose rates of a promising novel biostimulant (“SB”, hereinafter) on cotton plants. Currently under development by Sofbey SA, Mendrisio, Switzerland, “SB” formulation is based on the Glicoligno Lipidic Complex, obtained through a company-developed methodology. Glicoligno Lipidic Complex can be defined as a mixture comprising an aliphatic lipidic fraction, a carbohydrate fraction, and an aromatic fraction, which were characterized by NMR, on the antioxidant metabolism, photosynthetic and agronomic parameters, and boll yield of cotton plants. We hypothesized that foliar application of the Glicoligno Lipidic Complex enhances cotton physiological performance and stress resilience under adverse climatic conditions, resulting in improved agronomic traits and increased yield. Such studies are essential to support sustainable cotton production and meet the growing global demand for natural fibers, particularly in regions frequently exposed to climatic stress, such as central Brazil.

## 2 Materials and methods

### 2.1 Site description

**Figure 1:**
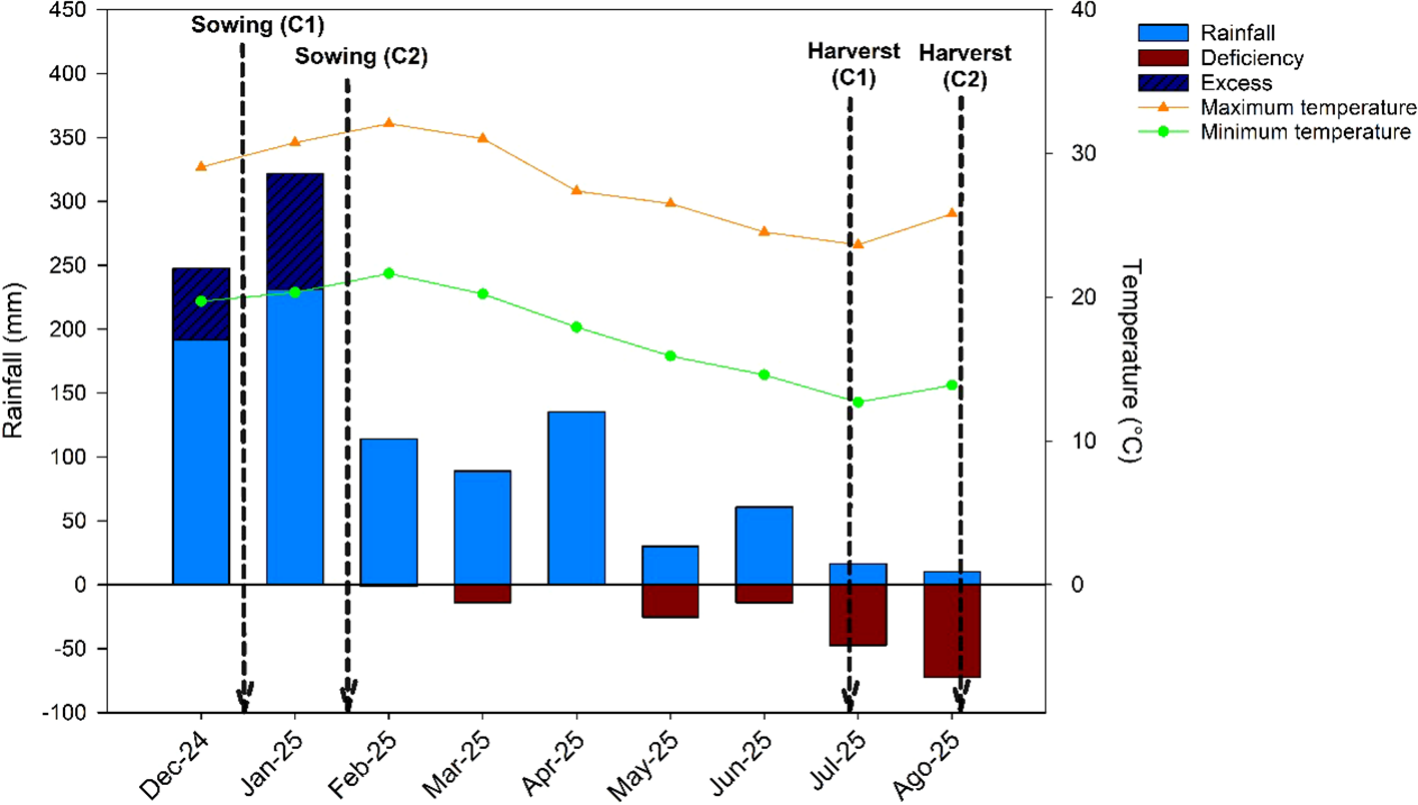
Average temperature and rainfall during the 2024/2025 cropping season at the experimental site in Botucatu, São Paulo, Brazil.

The study was conducted during the 2024/2025 cropping season under field conditions in Botucatu, São Paulo, Brazil (22°59’38.50”S, 48°30’2.09”W, elevation 851 m). The climate is Cwa according to Köppen’s classification, corresponding to humid subtropical with rainy summers and dry winters (Alvares et al., 2013). During the experimental period, meteorological data, including rainfall and maximum and minimum air temperatures, were recorded by an automatic weather station located near the experimental site. Reference evapotranspiration (ET_0_) was estimated using the Penman–Monteith method (Allen et al., 1998). Cotton crop evapotranspiration (ETc) was calculated by applying the appropriate crop coefficient (K_c_) for each phenological stage (Allen et al., 1998). Rainfall data were also used to assess the climatological water balance, which was determined by an electronic spreadsheet (Rolim et al., 1998) following the procedure for estimating actual evapotranspiration (ET_r_) (Thornthwaite and Mather, 1995). The precipitation, humidity, and temperature profiles are presented in **Figure 1**.

The soil texture in the experimental area is sandy loam. According to the Brazilian Soil Classification System (Santos et al., 2018). The soil is classified as a dystrophic Red Latosol, equivalent to a Haplorthox in the USDA soil taxonomy (Soil Survey Staff, 2014). Prior to the start of the experiment, physical analyses of the 0–0.20 m and 0.20–0.40 m layers, including particle size determination (Donagema et al., 2017), and soil fertility chemical analyses (van Raij et al., 2001), were performed. The results of these analyses are presented in **Table 1**.

**Table 1:** Physicochemical properties of the soil at the experimental site in Botucatu - SP.

Soil properties		Unit	0-0.20 m	0.20-0.40 m
Particle size				
Clay		g dm^-3^	191	230
Silt		g dm^-3^	101	112
Sand		g dm^-3^	708	658
Chemical attributes				
pH (CaCl_2_)		–	6.20	5.40
Soil Organic Matter (MOS)		g dm^-3^	18	13
Available Phosphorus (P _resin_)		mg dm^-3^	32	14
Available	Calcium (Ca^2+^_resin_)	mmol_c_ dm^-3^	53	19
	Magnesium (Mg^2+^_resin_)	mmol_c_ dm^-3^	17	6
	Potassium (K^+^_resin_)	mmol_c_ dm^-3^	1.90	1.20
	Aluminum (Al^3+^_KCl_)	mmol_c_ dm^-3^	0	0
Potential Acidity (H+Al)		mmol_c_ dm^-3^	17	21
S-Sulfate (S–SO_4_^2-^_Ca(H2PO4)2_)		mg dm^-3^	6	9
Boron (B hot water)		mg dm^-3^	0.18	0.12
Copper (Cu _DTPA-TEA_^a^)		mg dm^-3^	1.30	0.98
Iron (Fe _DTPA-TEA_)		mg dm^-3^	33	27
Manganese (Mn _DTPA-TEA_)		mg dm^-3^	2.80	1.70
Zinc (Zn _DTPA-TEA_)		mg dm^-3^	1.20	0.50
Base Saturation (V%)		%	80.88	55.51
Cation Exchange Capacity (CTC _pH 7.0_)		mmol_c_ dm^-3^	88.90	47.20

^a^ DTPA-TEA, diethylenetriaminepentaacetic acid-triethanolamine.

The cotton cultivars used in the experiment were FM 911GLTP (C_1_), and FM974 GLT (C_2_). FM 911GLTP (FiberMax^®^, BASF) is a medium-cycle cultivar (140–160 days) carrying the GLTP technology package (GlyTol^®^ + LibertyLink^®^ + TwinLink Plus^®^), which provides tolerance to glyphosate and glufosinate herbicides and resistance to major lepidopteran pests, such as cotton bollworm and leaf miner. The cultivar has modern plant architecture, large and heavy bolls (~5.4 g), average lint yield above 40%, fiber length around 29–30 mm, micronaire values between 4.1 and 4.6, and high strength. FM 911GLTP is resistant to bacterial blight (*Xanthomonas citri pv. malvacearum*) and shows broad adaptation across the main Brazilian cotton-producing regions.

FM 974 GLT is a genetically modified cultivar that combines high productivity with resistance to pests and diseases. Developed by BASF, FM 974 GLT is a medium-cycle variety (approximately 140–160 days) that also has the GLTP technology package (GlyTol^®^ + LibertyLink^®^ + TwinLink Plus^®^). This variety has a modern and robust plant architecture, with high-quality fibers and good resistance to climatic stress. It features high fiber yield with fiber length of around 28–30 mm, micronaire of 3.5-4.5, and good fiber strength. It is also resistant to bacterial blight (*Xanthomonas citri pv. malvacearum*) and adapted to several cotton-producing regions in Brazil, such as Mato Grosso, Bahia, and Goiás.

### 2.2 Experimental design and treatments

The experiment was conducted using a randomized completely block design consisting of five treatments with four replicates each, resulting in a total of 20 experimental units. Each plot covered an area of 35 m^2^. Four replicates were chosen to ensure that the degrees of freedom for the residuals were maintained at a minimum of 10, thereby providing sufficient statistical accuracy for analysis, and has been successfully used in previous cotton studies (Cottee et al., 2010; Tepecik et al., 2022). Additional crop management information is provided in **Table 2**.

**Table 2:** Experimental management timeline for cotton cultivars.

Management	Cultivar description	
	FM974 GLT (C_2_)	FM 911GLTP (C_1_)
Spacing	0.90 m	0.90 m
Plots	35 m^2^	35 m^2^
Replication	4	4
Sowing	16/12/2024	16/01/2025
Emergence of seedlings	22/12/2024	28/01/2025
Topdressing fertilization	24/01/2025	04/03/2025
F_1_ flowering	01/02/2025	03/05/2025
Harvest	26/06/2025	01/08/2025

**Figure 2:**
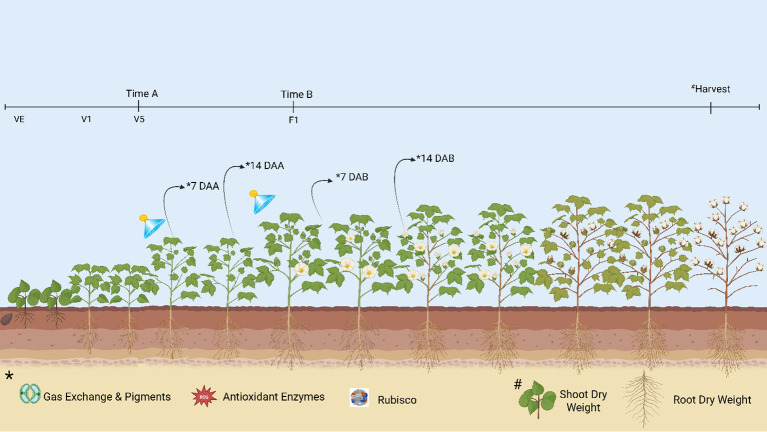
Schematic experimental timeline indicating phenological stages, timing of biostimulant application, and variable analysis. Biostimulant applications were performed at Time A (V_5_ stage) and Time B (F_1_ stage). Measurements were taken 7 and 14 days after application A (7 DAA and 14 DAA) and 7 and 14 days after application B (7 DAB and 14 DAB). VE = emergence; V_1_ = first leaf; V_5_ = fifth leaf; F_1_ = first flower. Variables measured include gas exchange and pigments, antioxidant enzymes, RuBisCO activity, shoot dry weight, and root dry weight (root icon).

The treatments consisted of a control and four different application doses of SB formulation, which is based on the Glicoligno Lipidic Complex, and can be defined as a mixture comprising an aliphatic lipidic fraction, a carbohydrate fraction, and an aromatic fraction, as shown in **Table 3**. Two foliar applications were performed: the first at the V_5_ phenological stage (Time A; 35–40 days after emergence), corresponding to five fully developed leaves, and the second at the F_1_ phenological stage (Time B; 55–65 days after emergence), when the first flower on the first reproductive branch bloomed. These phenological stages were chosen to coincide with key periods of vegetative and early reproductive development, when plants are particularly responsive to foliar nutrition and stress mitigation strategies. The doses evaluated in this study were selected and determined based on a previous study (Moretti et al., 2026a). The experimental timeline is illustrated in **Figure 2**.

**Table 3:** Experimental treatments for cotton.

Treatment		Application dose (L ha^-1^)	Application timing
1	Control	only water	
2	SB 0.5	0.50	A;B
3	SB 1.0	1.00	A;B
4	SB 1.5	1.50	A;B
5	SB 2.0	2.00	A;B

The applications followed common local practices and the recommended plant protection product program, avoiding the use of other products in the tank besides SB. The product was mixed in water no more than two hours before application according to the specific dose for each treatment and applied in a total application volume of 100 L ha^-1^. The cotton was harvested once the crop reached commercial maturity.

### 2.3 Assessments

#### 2.3.1 Root biometrics

The root systems of selected plants were extracted and carefully washed to remove soil particles while preserving root integrity (Moretti et al., 2020). The roots were then scanned using the WinRhizo image analysis system to determine root biometrics, including total root length, surface area, and volume. For scanning, the roots were placed without overlapping in a transparent water-filled tray on the scanner bed. After scanning, the roots were dried in an oven at 60–70 °C for 48–72 h until a constant weight was achieved and subsequently weighed using an analytical balance to determine root dry weight.

#### 2.3.2 Photosynthetic pigments

To determine the leaf contents of the photosynthetic pigments chlorophyll *a*, chlorophyll *b*, total carotenoids and total chlorophylls, five discs with a diameter of 0.5 cm were cut from the last fully expanded leaf, between the edge and the central vein. The leaf samples were stored for 24 h in 2 mL of N,N-dimethylformamide (DMF) in glass vials wrapped in aluminum foil (Lichtenthaler, 1987). Pigment contents were quantified spectrophotometrically at wavelengths of 664, 647 and 480 nm for chlorophylls *a* and *b* and carotenoids, respectively (Wellburn, 1994).

*Gas Exchange and Photosynthetic Pigments*

An infrared gas analyzer (IRGA, model CIRAS-3, PP Systems) was used to determine the net photosynthetic rate (A) (μmol m^-2^ s^-1^ stomatal conductance (*gs*) (mol m^-2^ s^-1^), carbon concentration in the substomatal chamber (Ci) (μmol mol^-1^), transpiration (E) (mmol m^-2^ s^-1^), carboxylation efficiency (A/Ci) and water use efficiency (A/E; WUE). For cotton, the third fully expanded leaf, counting from the apex to the base, was sampled from 5 plants per plot. All evaluations were performed in the morning, between 9 a.m. and 11 a.m., with constant ambient CO_2_ of 390 μmol mol^-1^.

#### 2.3.3 Oxidative stress and antioxidant metabolism

The concentration of hydrogen peroxide (H_2_O_2_) was determined according to the methodology proposed by Alexieva et al. (2001). Frozen plant material was ground with a mortar and pestle under liquid nitrogen, and 0.1% trichloroacetic acid (TCA) was added as an extraction buffer. The homogenized material was centrifuged, and an aliquot of the supernatant was mixed with 100 mM potassium phosphate buffer (pH 7.5) and 1 M potassium iodide and incubated at 1 °C for 1 h. After warming to room temperature, readings at a wavelength of 390 nm were obtained with a spectrophotometer. The concentration of H_2_O_2_ in the leaf was determined by reference to a standard curve and expressed in µmol g^-^¹ fresh weight.

Lipid peroxidation was assessed by determining leaf MDAcontent according to the methodology proposed by Heath and Packer (1968). Extraction was performed using 0.4 g of frozen plant material ground with a mortar and pestle under liquid nitrogen and suspended in 0.1% (w/v) TCA + 20% (w/v) polyvinylpolypyrrolidone (PVPP). The homogenized material was centrifuged. An aliquot of the supernatant was mixed with a solution of 20% TCA + 0.5% thiobarbituric acid (w/v) to initiate the reaction. The reactions were heated at 95 °C for 30 min and then placed on ice. Readings at wavelengths of 535 nm and 600 nm were obtained with a spectrophotometer. The results were expressed as nmol MDA g^-^¹ fresh weight.

The activities of superoxide dismutase (SOD, EC: 1.15.1.1) and catalase (CAT, 1.11.1.6) in cotton leaves were assessed to evaluate antioxidant metabolism. SOD activity was determined according to Giannopolitis and Ries (1977). The reaction was carried out in a reaction chamber under illumination with a 15 W fluorescent lamp at 25 °C after adding 50 mM potassium phosphate buffer pH 7.8, 13 mM methionine, 75 mM nitro blue tetrazolium (NBT), 0.1 mM EDTA, and 2 μM riboflavin to the protein extract described above. A control was prepared for each sample under the same conditions, except that the tubes were covered with aluminum foil to prevent light exposure. After 15 min, the samples were vortexed, and readings at a wavelength of 560 nm were obtained with a spectrophotometer.

CAT activity was determined by monitoring the degradation of H_2_O_2_ according to the methodology proposed by Azevedo et al. (1998). An aliquot of protein extract was added to a mixture containing 100 mM potassium phosphate buffer pH 7.5 and 30% (v/v) H_2_O_2_ in a test tube and quickly mixed by vortexing. The enzyme activity was determined by the decomposition of H_2_O_2_ over a 2-min interval, measured at a wavelength of 240 nm by spectrophotometry.

#### 2.3.4 Biometric assessments and grain yield

The useful area of each cotton plot (2 m in 2 central rows) was harvested manually, and the final cotton plant population (number of plants in the four central rows, excluding 1 m from the end of each row and extrapolated to plants per hectare), plant height, number of fruiting stems, and number of bolls per plant were measured. Additionally, the boll mass and plume and seed productivity were measured and converted to kg ha^-1^. The seeds were separated from the plumes to determine quality parameters, including uniformity, short fiber index, strength, micronaire, maturity, elongation, reflectance, and yellowness. The evaluations were carried out with the aid of a High-Volume Instrument (HVI).

### 2.4 Statistical analysis

The data were first subjected to error normality analysis (Shapiro and Wilk, 1965) and homoscedasticity of variances (Levene, 1960). After confirming that these assumptions were met, the data were subjected to analysis of variance (ANOVA) with an F-test applied at a probability level of *p* ≤ 0.10. Means were compared using Fisher’s protected t-test at *p* ≤ 0.10.

## 3 Results

### 3.1 Photosynthetic pigment contents and RuBisCO activity in cotton leaves

Foliar SB application enhanced cotton leaf photosynthetic pigment content and RuBisCO activity (**Table 4**). Across both cultivars, chlorophyll *a* content increased (*p* ≤ 0.05) with SB dose, peaking at SB 2.0, with gains of 13.2% in C_1_ and 22.4% in C_2_, and remained elevated across application times. Chlorophyll *b* showed limited variation, except for a 19.6% increase (*p* ≤ 0.05) in C_2_ at 7 DAA with SB 2.0. while Total chlorophyll followed similar patterns, with maximum increases (*p* ≤ 0.05) of 25.5% in C_2_ at 7 DAA (*p* ≤ 0.01), remaining 13.5% higher at 14 DAA.

**Table 4:** Concentrations of photosynthetic pigments (chlorophyll a – Chl *a*; chlorophyll b – Chl *b*; total chlorophyll – Chl t; and total carotenoids – Car t) and RuBisCO activity in diagnostic leaves of two cotton cultivars (C_1_ and C_2_) as a function of foliar SB application during the 2024/2025 growing season.

Treatment	Chl *a*		Chl *b*		Chl t		Car t		RuBisCO	
	–––––––––––––––––mg g^-1^ fresh weight –––––––––––––––––								µmol CO_2_ g^-^¹ DW h^-^¹	
	C_1_	C_2_	C_1_	C_2_	C_1_	C_2_	C_1_	C_2_	C_1_	C_2_
Time A										
7 DAA at the V_5_ phenological stage										
Absolute Control	797c **	945c	397a	397c	1194b	1343c	455b	465a	26.8a	32.1c
SB 0.5 L	870ab	1004bc	423a	462ab	1292a	1466b	465b	491a	28.9a	34,4bc
SB 1.0 L	835bc	1071ab	443a	433bc	1278a	1504b	503ab	484a	31.8a	37.4ab
SB 1.5 L	884ab	1149a	430a	448ab	1314a	1596a	533a	495a	27.7a	38.2ab
SB 2.0 L	902a	1157a	433a	475a	1335a	1632a	528a	514a	29.0a	39.7a
p-value	0.0306 *	0.0037	0.2639	0.0226	0.0248	0.0009	0.0485	0.3545	0.2452	0.0753
C.V. (%)	4.94	6.44	6.72	6.60	4.13	4.79	7.95	6.54	10.4	10.0
14 DAA at the V_5_ phenological stage										
Absolute Control	813c	1069b	411a	510a	1224b	1579a	472c	482c	28.5b	32.5a
SB 0.5 L	910ab	1074b	439a	528a	1350a	1602a	482c	504bc	30.9a	32.8a
SB 1.0 L	876b	1155ab	462a	497a	1338a	1652a	525b	528ab	31.2a	33.2a
SB 1.5 L	928ab	1174a	447a	485a	1376a	1659a	558a	538ab	31.4a	34.2a
SB 2.0 L	944a	1188a	451a	476a	1395a	1665a	552ab	547a	31.2a	35.2a
p-value	0.0111	0.0990	0.4031	0.5805	0.0289	0.3847	0.0003	0.0668	0.0470	0.1057
C.V. (%)	5.06	6.34	8.30	9.45	5.03	4.41	4.25	6.01	4.36	4.26
Time B										
7 DAB at the F_1_ phenological stage										
Absolute Control	780b	1059b	428a	488a	1208b	1547a	488a	507a	27.1c	33.8bc
SB 0.5 L	814ab	1157a	430a	485a	1244ab	1642a	482a	538a	28.1bc	33.0c
SB 1.0 L	840a	1176a	426a	510a	1266ab	1686a	496a	527a	29.9ab	35.1bc
SB 1.5 L	855a	1176a	438a	481a	1293a	1657a	507a	540a	29.4abc	35.9b
SB 2.0 L	862a	1198a	416a	489a	1288a	1687a	516a	541a	31.1a	39.6a
p-value	0.0651	0.0710	0.8675	0.8006	0.2833	0.1126	0.3179	0.5469	0.0945	0.0125
C.V. (%)	4.68	5.62	6.59	7.04	4.62	4.56	4.86	6.00	6.70	6.37
14 DAB at the F_1_ phenological stage										
Absolute Control	721a	1020b	455a	458a	1176b	1478c	341a	483b	27.3b	31.4c
SB 0.5 L	761a	1073b	454a	488a	1215ab	1561b	357a	510a	29.8a	32.6bc
SB 1.0 L	770a	1131a	457a	477a	1227ab	1608ab	338a	512a	29.8a	34.1ab
SB 1.5 L	786a	1165a	470a	469a	1256a	1634a	342a	523a	30.3a	35.0a
SB 2.0 L	809a	1179a	460a	479a	1269a	1658a	336a	520a	31.4a	35.4a
p-value	0.1265	0.0018	0.8454	0.3692	0.0879	0.0007	0.9079	0.0094	0.0122	0.0310
C.V. (%)	5.70	4.13	4.64	4.39	3.69	2.79	9.72	2.67	4.51	5.08

*Data were analyzed by analysis of variance (ANOVA), and when significant differences were identified, means were compared using Fisher’s protected least significant difference (LSD) test at *p* < 0.10. **Columns with different letters are significantly different according to the LSD test (*p* < 0.10).

Carotenoids increased (*p* ≤ 0.05) in C_1_ at 7 DAA (16.6% in SB 1.5 and 2.0) and at 14 DAA for both cultivars (11.6% in C_1_, 15.1% in C_2_). After the second application, significant increases (*p* ≤ 0.05) were observed only in C_2_ at 14 DAB (5.16% over control).

RuBisCO activity showed consistent enhancement across cultivars and time points. SB 2.0 induced the highest increases (*p* ≤ 0.1) at 7 DAA/DAB (14.8–22.3%), while later measurements in C_1_ were enhanced (*p* ≤ 0.05) by 9.5–11.1% and in C_2_ by 11.5–12.7% with SB 1.5 and 2.0.

Overall, SB application improved photosynthetic pigments and RuBisCO activity consistently across time points and cultivars, demonstrating a clear positive effect on cotton photosynthetic efficiency.

### 3.2 Gas exchange parameters in cotton leaves

Net photosynthesis (A) increased significantly with Glicoligno Lipidic Complex application across both cultivars. At 7 DAA, average gains were 14.4% compared with the control (*p* ≤ 0.05). At 14 DAA, the maximum increase was observed in C_1_ with the 2.0 L ha^-^¹ dose (12.8%; p ≤ 0.05), whereas C_2_ responded significantly to all doses, with an average increase of 6.3% (*p* ≤ 0.05). These increases persisted at 7 DAB, reaching 11.5% in C_1_ and 15.0% in C_2_ (*p* ≤ 0.05), and at 14 DAB, when C_1_ exhibited higher A in all treatments and C_2_ showed average gains of 10.8% at the three highest doses (*p* ≤ 0.05) (**Table 5**).

**Table 5:** Net photosynthetic rate (A − µmol CO_2_ m^-^² s^-^¹), stomatal conductance (gs − mol H_2_O m^-^² s^-^¹), intercellular CO_2_ concentration (Ci − µmol CO_2_ mol^-^¹), leaf transpiration rate (E − mmol H_2_O m^-^² s^-^¹), water use efficiency (WUE − μmol CO_2_ (mmol H_2_O)^-^¹), and carboxylation efficiency (CE − dimensionless) in diagnostic leaves of two cotton cultivars (C_1_ and C_2_) as a function of foliar SB application during the 2024/2025 growing season.

Treatments	A		gs		Ci		E		WUE		CE	
	C_1_	C_2_	C_1_	C_2_	C_1_	C_2_	C_1_	C_2_	C_1_	C_2_	C_1_	C_2_
Time A												
7 DAA at the V_5_ phenological stage												
Absolute Control	11.8c**	11.5c	0.22bc	0.21b	211a	211a	3.12a	3.09a	3.79c	3.73b	0.056d	0.055d
SB 0.5 L	12.7bc	11.8bc	0.22abc	0.22ab	206ab	209ab	3.06a	3.13a	4.04bc	3.77b	0.060cd	0.057cd
SB 1.0 L	12.6bc	12.3ab	0.21c	0.21b	203bc	206b	2.93a	3.00a	4.30abc	4.09a	0.062bc	0.060bc
SB 1.5 L	13.0ab	12.7a	0.22ab	0.22a	196cd	201c	2.97a	3.00a	4.40ab	4.25a	0.066ab	0.064a
SB 2.0 L	13.5a	12.6a	0.23a	0.22a	193d	200c	2.87a	2.99a	4.74a	4.21a	0.070a	0.063ab
p-value	0.0454*	0.0570	0.0568	0.0349	0.0018	0.0003	0.2894	0.1612	0.0559	0.0070	0.0038	0.0039
C.V. (%)	5.59	4.71	3.11	2.16	2.52	1.37	5.71	2.98	9.56	4.99	6.51	4.81
14 DAA at the V_5_ phenological stage												
Absolute Control	12.5d	12.7b	0.21a	0.22a	203a	201a	3.37a	2.93a	3.72c	4.35a	0.062c	0.063a
SB 0.5 L	13.1c	13.3a	0.21a	0.22a	195a	202a	3.32a	3.13a	3.97bc	4.26a	0.067bc	0.066a
SB 1.0 L	13.3bc	13.5a	0.21a	0.22a	187a	202a	3.17a	2.93a	4.20ab	4.63a	0.072ab	0.067a
SB 1.5 L	13.8ab	13.5a	0.22a	0.23a	189a	200a	3.21a	3.00a	4.31a	4.52a	0.073a	0.068a
SB 2.0 L	14.1a	13.8a	0.22a	0.22b	186a	200a	3.17a	2.93a	4.46a	4.85a	0.076a	0.069a
p-value	0.0023	0.0468	0.3071	0.4893	0.1954	0.6880	0.3288	0.1846	0.0120	0.2167	0.0034	0.1278
C.V. (%)	3.29	3.51	3.86	2.67	5.43	1.69	5.06	5.00	6.23	7.82	5.82	4.46
Time B												
7 DAB at the F_1_ phenological stage												
Absolute Control	13.1c	12.0b	0.23bc	0.23a	208a	209a	4.14a	3.08a	3.17c	3.91b	0.063d	0.058b
SB 0.5 L	13.6bc	11.7b	0.23abc	0.22a	202ab	201a	4.09a	2.93a	3.36bc	4.03b	0.068c	0.058b
SB 1.0 L	13.8b	13.6a	0.22c	0.22a	195bc	202a	3.95a	2.95a	3.52ab	4.61a	0.071bc	0.067a
SB 1.5 L	14.5a	13.6a	0.23ab	0.22a	193c	203a	3.99a	2.93a	3.69a	4.65a	0.075ab	0.067a
SB 2.0 L	14.7a	14.1a	0.23a	0.23a	190c	200a	3.91a	2.90a	3.76a	4.88a	0.077a	0.071a
p-value	0.0035	0.0104	0.0714	0.4726	0.0040	0.1691	0.2970	0.6326	0.0164	0.0360	0.0009	0.0110
C.V. (%)	3.66	7.18	2.82	2.28	2.68	2.60	4.08	5.79	6.39	9.99	5.22	8.13
14 DAB at the F_1_ phenological stage												
Absolute Control	12.8b	12.00b	0.21c	0.22a	192a	207a	4.28a	3.06a	3.00a	3.92b	0.068a	0.056a
SB 0.5 L	13.9a	12.2b	0.22b	0.22a	203a	204b	4.63a	3.06a	3.00a	4.00b	0.070a	0.055a
SB 1.0 L	14.1a	13.1a	0.22b	0.22a	206a	204b	4.34a	2.96a	3.26a	4.43a	0.066a	0.049b
SB 1.5 L	14.1a	13.3a	0.24a	0.22a	202a	201c	4.44a	2.98a	3.19a	4.47a	0.070a	0.050b
SB 2.0 L	14.0a	13.5a	0.24a	0.22a	207a	200c	4.39a	2.92a	3.21a	4.62a	0.066a	0.048b
p-value	0.0455	0.0013	0.0001	0.1340	0.1936	0.0057	0.6507	0.2500	0.1812	0.0011	0.4449	0.0011
C.V. (%)	4.30	3.48	2.81	1.18	4.47	1.08	7.54	3.41	5.70	4.76	3.40	4.61

*Data were analyzed by analysis of variance (ANOVA), and when significant differences were identified, means were compared using Fisher’s protected least significant difference (LSD) test at *p* < 0.10. **Columns with different letters are significantly different according to the LSD test (*p* < 0.10).

Stomatal conductance (gs) were significantly increased (*p* ≤ 0.05) at 7 DAA (4.5% in C_1_, 4.8% in C_2_) for SB treatments, but not at 14 DAA. After the second application, C_1_ showed increases of 4.5% and 14.2% at 7 and 14 DAB, respectively; C_2_ showed no significant change post-second application.

Intercellular CO_2_ concentration (Ci) decreased (*p* ≤ 0.1) most at 7 DAA with highest SB doses (7.5% in C_1_, 4.7% in C_2_), with no differences at 14 DAA. Post-second application, significant reductions occurred in C_1_ at 7 DAB (7.2%) and in C_2_ at 14 DAB (2.3%), while transpiration (E) was not significantly affected.

Water use efficiency (WUE) improved (*p* ≤ 0.05) with SB 1.0, 1.5, and 2.0 at 7 DAA (14.0% in C_1_, 12.1% in C_2_). At 14 DAA, only C_1_ showed a 16.1% increase. After the second application, WUE gains were 15.5% in C_1_ and 20.5% in C_2_ at 7 DAB, and 14.8% in C_2_ at 14 DAB for the three highest doses.

Carboxylation efficiency (CE) was highest with SB 1.5 and 2.0 at 7 DAA (21.4% in C_1_, 16.4% in C_2_). At 14 DAA, only C_1_ showed a significant (*p* ≤ 0.05) 21% increase. At 7 DAB, C_1_ reached 20.6% with SB 1.5 and 2.0, and C_2_ responded to these doses plus SB 1.0 (17.2%). At 14 DAB, CE in C_1_ did not differ among treatments, whereas in C_2_, the highest values were observed for control and SB 0.5, with no significant difference between them (**Table 5**).

### 3.3 Antioxidant responses in cotton leaves

Overall, cotton leaf H_2_O_2_ content was highest in the control and at lower SB doses. Across cultivars, SB application consistently reduced H_2_O_2_ levels, with C_1_ showing a 6.4% decrease (*p* ≤ 0.05) at 7 DAA for the three highest doses, 12.2% at 14 DAA for SB 1.5 and 2.0, and 10.8% at 7 DAB for SB 2.0. In C_2_, reductions were observed across all SB treatments, with an average decrease (*p* ≤ 0.05) of 7.6%, and at 14 DAB, the three highest doses produced decreases of 4.5% in C_1_ and 10.0% in C_2_ (**Table 6**).

**Table 6:** Levels of hydrogen peroxide (H_2_O_2_) and lipid peroxidation [malondialdehyde (MDA)] and activities of the antioxidant enzymes superoxide dismutase (SOD) and catalase (CAT) in diagnostic leaves of two cotton cultivars (C_1_ and C_2_) as a function of foliar SB application during the 2024/2025 growing season.

Treatments	H_2_O_2_		MDA		SOD		CAT	
	µmol g^-1^ fresh weight		nmol g^-1^ fresh weight		Units mg^-1^ protein		µmol min^-1^ mg^-1^ protein	
	C_1_	C_2_	C_1_	C_2_	C_1_	C_2_	C_1_	C_2_
Time A								
7 DAA at the V_5_ phenological stage								
Absolute Control	32.6a**	33.6a	15.3a	30.8a	154a	219a	0.30a	5.89a
SB 0.5 L	32.9a	32.4a	13.8b	29.5ab	142b	213a	0.29a	5.82a
SB 1.0 L	30.2b	32.6a	13.4b	28.8bc	144b	208a	0.27a	5.75a
SB 1.5 L	30.7b	32.9a	14.3ab	27.4c	142b	208a	0.27a	5.15b
SB 2.0 L	30.7b	32.4a	14.0b	27.5c	136c	205a	0.29a	5.13b
p-value	0.0080	0.3001	0.0534	0.0356	0.0012	0.1019	0.2937	0.0023
C.V. (%)	3.24	2.46	5.74	5.14	2.96	3.35	7.26	4.83
14 DAA at the V_5_ phenological stage								
Absolute Control	36.0a	31.5a	15.4a	25.4a	158a	211a	0.33a	5.52a
SB 0.5 L	34.0b	30.9a	15.0a	25.9a	142b	201a	0.29a	5.12a
SB 1.0 L	33.3b	30.3a	14.3b	24.6a	143b	199a	0.31a	5.11a
SB 1.5 L	31.4c	30.8a	14.0bc	25.2a	136c	196a	0.29a	5.01a
SB 2.0 L	31.8c	31.2a	13.7c	24.9a	137c	198a	0.27a	5.28a
p-value	0.0002	0.3290	0.0002	0.7218	0.0001	0.1374	0.3174	0.3613
C.V. (%)	3.01	2.36	2.71	5.66	2.89	3.90	11.8	6.91
Time B								
7 DAB at the F_1_ phenological stage								
Absolute Control	34.2a	28.8a	15.0a	12.5a	132a	181a	0.31a	3.32a
SB 0.5 L	33.4a	27.3b	14.1b	11.0b	126b	181a	0.28bc	2.94a
SB 1.0 L	31.7b	26.8b	13.8b	10.8b	127ab	177a	0.27bc	3.19a
SB 1.5 L	31.0bc	26.2b	13.9b	10.6b	126ab	183a	0.30ab	2.88a
SB 2.0 L	30.5c	26.1b	13.8b	10.8b	119c	181a	0.27c	3.17a
p-value	0.0002*	0.0185	0.0031	0.0197	0.0454	0.8549	0.0888	0.7569
C.V. (%)	2.65	3.86	2.54	6.79	4.00	4.18	7.87	17.3
14 DAB at the F_1_ phenological stage								
Absolute Control	35.3a	28.3a	16.1a	12.7a	116a	215a	0.31a	5.70a
SB 0.5 L	34.9ab	27.0ab	15.2ab	10.6b	113a	207ab	0.29ab	5.45ab
SB 1.0 L	34.1bc	25.5b	14.8b	10.5b	114a	203b	0.27bc	5.40abc
SB 1.5 L	33.9bc	25.5b	14.4b	12.0a	113a	202b	0.28abc	5.20bc
SB 2.0 L	33.6c	25.5b	14.2b	12.2b	110a	201b	0.25c	5.08c
p-value	0.0861	0.0856	0.0565	0.0128	0.5630	0.0868	0.0546	0.0576
C.V. (%)	2.52	5.76	5.64	8.85	4.84	3.31	8.05	5.06

*Data were analyzed by analysis of variance (ANOVA), and when significant differences were identified, means were compared using Fisher’s protected least significant difference (LSD) test at *p* < 0.10. **Columns with different letters are significantly different according to the LSD test (*p* < 0.10).

MDA content followed similar trends: at 7 DAA, all SB treatments except SB 1.5 reduced MDA in C_1_ by 10.5%, and in C_2_, the two highest doses decreased (*p* ≤ 0.05) MDA by 10.7%. At 14 DAA, C_1_ showed an 11% reduction with SB 2.0, while C_2_ showed no significant change. At 7 DAB, reductions in MDA were 7.3% in C_1_ and 13.6% in C_2_, and at 14 DAB, C_1_ experienced the greatest decrease (*p* ≤ 0.05) (14.5%), while C_2_ responded to all doses except SB 1.5.

SOD activity decreased (*p* ≤ 0.05) in C_1_ by 11.7% at 7 DAA with SB 2.0, and by an average of 13.6% at 14 DAA with SB 1.5 and 2.0. Post-second application, decreases were observed at 7 DAB in C_1_ and 14 DAB in C_2_. C_1_ reached the lowest SOD activity with SB 2.0 (9.8% decrease), while in C_2_, SB 1.0, 1.5, and 2.0 produced an average decrease of 6.0% (**Table 6**).

CAT activity did not differ significantly after the first application. After the second application, decreases (*p* ≤ 0.1) were observed only in C_1_ at 7 DAB, with SB 2.0 reducing activity by 12.9%. At 14 DAB, SB 2.0 decreased SOD activity by 19.4% in C_1_ and 10.9% in C_2_.

### 3.4 Fiber quality parameters

Foliar SB application had no significant effects on quality parameters with two exceptions in C_1_. First, SB 1.0 decreased C_1_ reflectance (*p* ≤ 0.05) by 4.1% compared to SB 0.5; however, the differences in reflectance between SB 0.5, SB 1.5, and the control were not significant. Second, SB 2.0 increased (*p* ≤ 0.05) yellowness by 8.8% compared to the control (**Table 7**).

**Table 7:** Fiber quality parameters in two cotton cultivars as affected by foliar SB application during the 2024/2025 growing season.

Treatment	UNF		SFI		STR		MIC		MAT		ELG		RD		+B	
	%		%		gf tex^-1^		µg pol^-1^		%		%		%		-	
	C_1_	C_2_	C_1_	C_2_	C_1_	C_2_	C_1_	C_2_	C_1_	C_2_	C_1_	C_2_	C_1_	C_2_	C_1_	C_2_
Absolute Control	84.8a	84.7a	8.48a	7.08a	31.5a	32.0a	4.03a	4.43a	0.88a	0.86a	7.63a	6.70a	47.8ab	48.4a	7.38b	7.15a
SB 0.5 L	85.1a	83.9a	7.43a	7.78a	31.5a	32.4a	4.28a	3.88a	0.90a	0.84a	7.33a	7.45a	48.5a	49.0a	7.30b	7.08a
SB 1.0 L	85.5a	83.8a	7.38a	7.88a	30.0a	30.1a	3.92a	4.18a	0.85a	0.85a	7.80a	7.15a	46.6c	49.0a	7.33b	7.20a
SB 1.5 L	84.9a	85.6a	8.05a	7.38a	31.0a	32.7a	4.23a	4.58a	0.88a	0.87a	7.43a	7.38a	47.8ab	48.5a	7.58b	7.65a
SB 2.0 L	85.1a	85.2a	7.56a	7.60a	31.4a	31.3a	4.10a	4.55a	0.88a	0.86a	7.60a	7.48a	47.0bc	48.1a	8.03a	7.65a
*p*-value	0.8767	0.3187	0.3761	0.5468	0.3693	0.1879	0.7287	0.1980	0.6991	0.1063	0.6244	0.3676	0.0268	0.1164	0.0468	0.3143
C.V. (%)	1.20	1.59	11.3	9.53	3.64	4.88	9.74	10.2	5.42	1.49	5.97	8.21	1.50	1.11	4.40	6.66

UNF (upper half mean length, %); SFI (short fiber index, %); STR (strength, gf tex^-^¹); MIC (micronaire, µg pol^-1^); MAT (maturity index, %); ELG (elongation, %); RD (reflectance, %); and +B (yellowness index).

*Data were analyzed by analysis of variance (ANOVA), and when significant differences were identified, means were compared using Fisher’s protected least significant difference (LSD) test at *p* < 0.10. **Columns with different letters are significantly different according to the LSD test (*p* < 0.10).

### 3.5 Cotton biometric parameters and yield components

Foliar application of SB did not significantly affect final plant population, root dry weight, or plant height in either cultivar, indicating that early vegetative growth and stand establishment were not influenced by the treatments (**Table 8**). In contrast, reproductive responses were more pronounced. In C_1_, SB 2.0 resulted in a significant increase (*p* ≤ 0.05) in the number of bolls per plant; however, this effect was statistically similar to that observed with SB 1.5, suggesting a plateau response between intermediate and high doses. In C_2_, no significant differences were detected for boll number per plant across treatments.

**Table 8:** Final plant population (FPP), root dry weight (RDW), plant height (PH), number of bolls per plant, boll weight (g) and fiber seed yield (kg ha^-1^) in two cotton cultivars as a function of foliar SB application during the 2024/2025 growing season.

Treatment	FPP		RDW		PH		Bolls				Fiber seed yield	
	ha^-1^ (x1000)		kg ha^-1^		cm		Number plant^-1^		g boll^-1^		kg ha^-1^	
	C_1_	C_2_	C_1_	C_2_	C_1_	C_2_	C_1_	C_2_	C_1_	C_2_	C_1_	C_2_
Absolute Control	94.0a	96.9a	792a	731a	112a	118a	8.75c	9.00a	4.44a	3.97c	4485b	3436b
SB 0.5 L	94.0a	93.8a	832a	740a	114a	121a	10.3b	9.18a	4.64a	4.08b	4660ab	3508b
SB 1.0 L	94.3a	94.4a	767a	740a	110a	119a	10.3b	9.15a	4.55a	4.12b	4695a	3554ab
SB 1.5 L	94.5a	94.4a	880a	751a	116a	118a	10.8ab	9.15a	4.67a	4.22a	4841a	3675a
SB 2.5 L	94.5a	95.6a	892a	754a	117a	123a	11.5a	9.26a	4.75a	4.21a	4851a	3695a
p-value	0.9999	0.9371	0.1539	0.7568	0.4567	0.5437	0.0018	0.8626	0.3452	0.0001	0.0297	0.0739
C.V. (%)	7.73	5.98	9.13	3.67	5.08	3.90	6.75	3.61	4.64	1.27	3.23	3.67

*Data were analyzed by analysis of variance (ANOVA), and when significant differences were identified, means were compared using Fisher’s protected least significant difference (LSD) test at *p* < 0.10. **Columns with different letters are significantly different according to the LSD test (*p* < 0.10).

Regarding yield components, boll weight (g boll^-^¹) was significantly affected only in C_2_ (*p* ≤ 0.05), where SB 1.5 and SB 2.0 increased boll weight by 6.3% and 6.0%, respectively, relative to the control, indicating a cultivar-dependent response in assimilate allocation or boll filling efficiency. Cotton yield (kg ha^-^¹) showed a consistent tendency to increase under the three highest SB doses in both cultivars; however, statistical significance was reached only for SB 2.0, which increased yield by 6.9% in C_1_ (*p* ≤ 0.05) and by 5.6% in C_2_ (*p* ≤ 0.1) compared with the control.

## 4 Discussion

Overall, the use of the biostimulant based on Glicoligno Lipidic complex provided vegetative and reproductive benefits to cotton plants. When applied as a foliar spray, this compound enhanced the plant’s antioxidant system, optimizing photosynthetic parameters. Consequently, greater efficiency in the distribution of fixed carbon resulted in increased productivity, without a direct effect on fiber quality (Bulgari et al., 2015). The attenuation of oxidative stress is particularly relevant in field conditions context, allowing plant metabolism to remain more efficient and capable of sustaining high productivity, even under the constant threat of adverse climatic conditions (Melo et al., 2024).

The long field cycle of cotton exposes the crop to intense climatic variations that can induce irreversible stress conditions, especially when grown as a second-season crop (mostly in Brazil) (Farooq et al., 2013; Aparecido et al., 2020). Overall, SB had positive effects on the control and reduction of oxidative stress parameters, helping to clear the cellular environment of ROS, particularly H_2_O_2_ (Moreira et al., 2026). H_2_O_2_ is commonly formed during aerobic metabolism and acts as a signal for stress-responsive genes, an antimicrobial agent, and an inducer of programmed cell death (Moretti et al., 2021; Rodrigues et al., 2025). However, excessive cellular levels of H_2_O_2_ harm overall metabolism by initiating lipid peroxidation of cellular membranes (Viveiros et al., 2024). Foliar SB application reduced the accumulation of not only H_2_O_2_ but also MDA, a three-carbon aldehyde that is the main byproduct of lipid peroxidation (Viveiros et al., 2025).

The positive effects of foliar SB application on oxidative stress may be related to improved photosynthetic performance and nutrient use, which likely reduced ROS generation and helped maintain cellular redox homeostasis, thereby lowering the demand for antioxidant enzyme activation. The biostimulant may have favored CO_2_ assimilation and physiological adjustment, limiting excess electron leakage to O_2_ and consequently reducing oxidative stress. In contrast to previous reports that fulvic acids stimulate antioxidant enzyme activity in maize (Anjum et al., 2011) and wheat (Ali et al., 2015). Our findings suggest that SB acted mainly by preventing upstream ROS formation rather than by enhancing the antioxidative system after stress establishment (Farooq et al., 2019; Tavanti et al., 2021). In addition, the maintenance of photosynthetic activity likely reduced cellular damage by decreasing the pool of free electrons available for radical formation (Rodrigues et al., 2021a).

Within the antioxidant system, SOD constitutes the first line of defense and catalyzes the dismutation of superoxide anion into H_2_O_2_ and molecular oxygen, preventing the formation of other ROS such as hydroxyl radical (OH), which is not enzymatically eliminated by plant metabolism (Rodrigues et al., 2025; Moretti et al., 2026b). The H_2_O_2_ generated by SOD dismutation is subsequently neutralized by CAT, which converts it into water and oxygen, thus contributing to proper cellular redox balance (Viveiros et al., 2024, 2025). Thus, the observed decreases in SOD and CAT activity are consistent with the overall reduction in oxidative stress, as indicated by lower H_2_O_2_ and MDA levels (Rossi et al., 2015).

Reducing oxidative stress leads to greater stability of thylakoids and antenna complexes, diminishing the degradation of chlorophylls and carotenoids within the photosynthetic apparatus. This alone would increase pigment accumulation in biostimulant-treated plants (Oliveira et al., 2022). Moreover, several amino acids found in biostimulants are direct precursors of chlorophyll biosynthesis and facilitate the absorption of essential nutrients due to their chelating action, such as magnesium, the central atom of the chlorophyll molecule (Rodrigues et al., 2021a; 2025).

The combination of reduced oxidative stress and optimized photosynthetic function likely supported RuBisCO stability and function in SB-treated plants. When the cellular environment is stable, RuBisCO synthesis, activation, and maintenance remain at consistent levels, contributing to potential improvements in crop productivity (Moreira et al., 2017; Jaghdani et al., 2021). RuBisCO is present in all photosynthetic organisms (Correia et al., 2020) and the increase in RuBisCO activity in response to foliar SB application to cotton plants reflects an increase in carbon assimilation (Moretti et al., 2026b). Optimization of the photosynthetic apparatus is indicated by the observed gains in net photosynthesis and stomatal conductance, driven by greater water use efficiency within the system. Conversely, transpiration and the carbon concentration in the substomatal cavity decreased under foliar SB application. The increase in RuBisCO activity is likely one of the main reasons for the increases in carbon assimilation and gas exchange efficiency, alongside the reduction in ROS and, consequently, potential abiotic stress (Bossolani et al., 2021; Moreira et al., 2022).

Foliar SB application enhanced the physiological adjustment of cotton under stress conditions. The maintenance of higher photosynthetic performance, together with stable transpiration rates, suggests that the biostimulant supported carbon assimilation while limiting water loss, thereby improving water use efficiency. Under water deficit, transpiration is regulated not only by stomatal behavior but also by a range of physiological and environmental factors, which may partly explain the variable responses reported across studies (Moreira et al., 2018; Moretti et al., 2018). Within this framework, previous studies indicate that while biostimulants can mitigate the physiological constraints imposed by stress by preserving photosynthetic activity, transpiration responses are not always consistent and may depend on product composition, crop species, and environmental conditions (Moreira et al., 2016; Santos et al., 2020).

Overall, foliar SB application had no significant effects on fiber quality parameters. The physiological improvements observed, including enhanced photosynthetic pigment content, increased RuBisco activity, improved gas exchange, and reduced oxidative stress (lower H_2_O_2_ and MDA levels), likely optimized carbon assimilation and resource allocation within the plant. These changes were preferentially directed toward vegetative and reproductive growth rather than fiber quality traits, which remained within ranges considered sufficient to excellent.

Comparable effects have been reported for dissolved organic matter substances, humic-based and microbial, particularly in maize, soybean, cassava and okra. These studied demonstrated improvements in root and shot growth, nutrient acquisition, and, in some cases, enhancement of photosynthetic and antioxidant metabolism (Fernández and Brown, 2013; Fernández et al., 2021). While not all previous studies evaluated the full range of physiological responses, our research highlights that SB uniquely ROS mitigation, maintenance of photosynthetic efficiency, and increased reproductive development in cotton, linking these biochemical effects directly to yield gains.

Finally, the positive effects on cotton yield can be mechanistically linked to these physiological adjustments. By maintaining higher photosynthetic efficiency and reducing oxidative damage, SB-treated plants were able to sustain energy and carbon flow toward the formation of reproductive structures (Ali et al., 2021). In C_1_, this translated into a higher number of bolls per plant, while in C_2_, the improvement in boll weight contributed to yield gains. The absence of significant changes in other growth variables suggest that SB primarily improved metabolic efficiency and carbon allocation, rather than increasing the size of individual reproductive organs. Thus, the observed physiological changes directly underpin the increased productivity, demonstrating a connection between the biochemical adjustments induced by SB agronomic outcomes.

## 5 Conclusion

Foliar application of the novel biostimulant SB (Glicoligno Lipidic complex) not only mitigated the effects of environmental stresses but also enhanced the physiological efficiency of cotton. The most pronounced effects were observed at higher doses, particularly at the maximum SB dose of 2.0 L ha^-1^, enhancing net photosynthesis (A) by up tp 15% and RuBisCo activity by 9-22%. Photosynthetic pigments were improved, with chlorophyll content increasing by up to 25% and carotenoids by up to 16% Oxidative stress markers, specifically H_2_O_2_ and MDA, were reduced by up to 12% and 14%, respectively, indicating alleviation of oxidative damage. These physiological improvements translated into higher reproductive success, with the number of bolls per plant increasing by 30% in C_1_ and boll weight increasing by 6-6.3% in C_2_. Overall, fiber seed yield increased by 6.9% in C_1_ and 5.6% in C_2_. By mitigating oxidative stress and optimizing carbon allocation, SB facilitated greater energy investment into vegetative and reproductive growth, ultimately increasing crop productivity. Thus, these results reinforce the potential of SB as a foliar biostimulant to enhance photosynthetic efficiency, stress tolerance, and crop yield. Further investigations are needed to elucidate in greater detail the mechanisms by which foliar SB application promotes these physiological and agronomic improvements.

## Data Availability

The raw data supporting the conclusions of this article will be made available by the authors, without undue reservation.
